# Exploring the impact of digital economy on urban entrepreneurship: Evidence from China’s cities

**DOI:** 10.1371/journal.pone.0307855

**Published:** 2024-07-30

**Authors:** Jiafeng Gu

**Affiliations:** Institute of Social Science Survey, Peking University, Beijing, China; Qingdao University, CHINA

## Abstract

This paper aims to examine the impact of the digital economy on urban entrepreneurship and its spatial spillover effects. To achieve this purpose, this research relies on data from 252 prefecture-level cities in China from 2012 to 2019. The findings demonstrate that the development of the digital economy has a positive influence on entrepreneurial activity in cities, with particularly effects observed robust at higher quantile levels. Additionally, the results suggest that urban entrepreneurial activity may be a siphoning effect, impeding entrepreneurship in neighboring cities. Furthermore, further investigation shows regional and policy heterogeneity.

## 1. Introduction

Entrepreneurship plays an important role in achieving the goal of full employment and promoting economic growth worldwide [1, 2]. By combining resources and factors of production, entrepreneurs create higher-value forms that enhance social wealth and promote economic growth [3, 4]. However, successful entrepreneurship is challenging as it involves high risk and lower success rates, which can reduce potential entrepreneurs [5]. In the face of increasing global economic uncertainty, it is crucial to explore the effective strategies to enhance entrepreneurial activity. This topic warranties in-depth study and analysis.

In recent years, the digital economy has experienced rapid growth, with its gross domestic product (GDP) of major countries increasing [6, 7]. The digital economy is deeply integrated into various fields of the economy and society, leveraging its characteristics of high permeability, rapidity, and external economies. This integration constantly generates new opportunities for industrial development and provides valuable information and technological support for entrepreneurship [8].Therefore, the relationship between the digital economy and entrepreneurship has become a topic of significant interest for governments, academia, and industry. Existing literature generally recognizes the digital economy’s role in promoting entrepreneurship [9–11]. By integrating information technology, big data, and artificial intelligence, the digital economy facilitates the emergence of new business models and expands channels for entrepreneurial activities [12, 13]. However, there is a lack of literature exploring the specific circumstances under which the digital economy effectively promotes entrepreneurship.

Entrepreneurial activities often exhibit spatial spillover effects. Existing literature emphasizes on the positive spatial spillover effects of entrepreneurial activities [14, 15]. Most studies support the knowledge spillover theory of entrepreneurship [16]. However, there are also studies that demonstrate negative spatial spillovers from entrepreneurial activity, known as the siphoning effect [17–19]. These findings have led to a reconceptualization and rethinking of the knowledge spillover theory of entrepreneurship [14]. However, the existing literature does not sufficiently explain this siphoning effect, of entrepreneurial activity has not been adequately explained in, and further exploration is needed to understand the underlying mechanisms.

To address the deficiencies in the existing literature, this study aims to explores the impact of the digital economy on urban entrepreneurial activities. It does so by constructing and estimating a spatial panel quantile regression model using panel data from 252 prefecture-level cities. The aim is to accurately determine the relationship between the digital economy and urban entrepreneurial activities, as well as the spatial spillover effects.

Specifically, the research questions are as follows:

RQ1. Under what circumstances does a fast-growing digital economy promote urban entrepreneurship?

RQ2. How does entrepreneurship development in one city affect entrepreneurship development in its neighboring cities?

Exploring these questions can provide some empirical support for urban economic development and urban entrepreneurship planning at the same time, so it is of great practical significance to explore the relationship between the digital economy and urban entrepreneurship in depth.

It is of great value and practical significance to carry out this research with Chinese cities as the object of study. Since the reform and opening up, China’s economy has risen to second place in the world. As economic development has gradually entered a new normal, China is faced with the dual challenges of insufficient momentum for domestic economic growth and uncertainty in the international environment. During the period of economic development transition, raising employment levels and promoting industrial structure transformation and upgrading are key aspects of promoting high-quality economic development. Entrepreneurship is one of the most important ways to realize the goal of full employment and promote economic growth, and the degree of entrepreneurial activity in a city is a key indicator of the degree of entrepreneurial activity in a region or city. As an important driving force for high-quality economic development, urban entrepreneurial activity plays an important role in promoting technological innovation, increasing employment opportunities, and facilitating the transformation and upgrading of industrial structure in cities. Meanwhile, along with the continuous integration of digital technologies such as mobile Internet, big data, artificial intelligence, blockchain and other digital technologies with the traditional economy, the scale of China’s digital economy and its share in the national economy have both been rapidly increased. This provides objective conditions for scientifically assessing the impact of the digital economy on urban entrepreneurship. There is no doubt that China’s experience in this regard can provide meaningful lessons and references for developing countries in general.

The following contents of this paper are structured as follows. Section 2 presents the literature review and hypothesis development. Section 3 provides the data, variables, and methods. The empirical results are provided in Section 4. Section 5 contains the discussion. The final section concludes the paper.

## 2. Literature research and hypothesis development

Digital economy is an emerging economic form based on digital technology [20]. It is defined as an economy influenced by on the development of the Internet and e-commerce [21]. Although there is no unified consensus on the specific connotations of the digital economy [22], it is generally recognized that it possesses characteristics such as innovative, low-cost, and resource-saving. However, there are potential challenges in the development of the digital economy. Although the total amount of information infrastructure construction has developed rapidly, there remains an imbalance in the development of the digital economy between regions due to disparities in information infrastructure construction [23]. Unfortunately, there is a lack of relevant and systematic research on how this pattern of development in the digital economy affects entrepreneurship.

Urban entrepreneurial activity refers to the entrepreneurial activity in cities, in contrast to rural entrepreneurial activity which takes place in rural areas [24]. In this study, urban entrepreneurial activity refers to the entrepreneurship of various types of owners within urban spatial complexes, based on previous studies [25, 26]. The existing literature has examined various factors influencing entrepreneurial activity in cities. These factors include government policies [27], labor market [28],smart cities construction [29], consumption [30], transportation [25], and technological innovation [31]. Regarding research methods for studying entrepreneurship, quantile regressions has been utilized in several studies [32–34]. However, there is a lack of literature focusing on the study of urban entrepreneurial activity that employs this approach to fine-tune research.

It is generally accepted that a positive relationship exists between the digital economy and entrepreneurship. The rapid development of the digital economy, including the information industry and e-commerce provides impetus for entrepreneurial activities [35]. The growth of the digital economy expands the sources and channels of information, enabling entrepreneurs to acquire information quickly and make informed decisions quickly [36]. This combination of the digital economy and entrepreneurial activity has given rise to digital entrepreneurship [37], with the digital economy providing conditions for digital entrepreneurship and the emergence of female digital entrepreneurs [9]. Moreover, the digital economy helps to foster an innovation spirit in entrepreneurship [11], which is beneficial for entrepreneurship. The digital economy boosts rural entrepreneurship [38], and stimulate entrepreneurship in emerging economies [13]. Based on the above discussion, the following hypotheses is proposed:

**H1.** The digital economy has a positive impact on entrepreneurial activity.

Entrepreneurial activities tend to generate spatial spillovers, wherein, entrepreneurial activity in one province can have positive effects on neighboring provinces. These spillover effects contribute to the efficiency of green innovation [39], and promote technological innovation in neighboring regions [15]. Entrepreneurial activity in one region can even boost the economic output of neighboring regions [40]. However, the knowledge spillover theory of entrepreneurship has also faced criticism. The knowledge spillover theory of entrepreneurship should not be exaggerated. The knowledge spillover theory is not effective in explaining entrepreneurial activity in U.S. firms [14]. These findings indicate the need to explore alternative theories to better understand the dynamic of entrepreneurial activity

It is evident that spatial spillovers from entrepreneurial activity can have both positive or negative effects [41]. While positive spillovers are often emphasized, there is evidence suggesting that entrepreneurial clusters and localized competition can lead to negative spatial spillover. A study tracking 88 counties in Ohio from 2002 to 2006 found that entrepreneurial clusters had a negative impact on startup rates in neighboring counties [17]. Similarly, a study focusing on 121 counties in Colorado and California 1990 to 2000 revealed that localized competition negatively affected entrepreneurial activity through spatial spillovers [18]. Another study examining 64 low- and middle-income countries and 25 high-income countries from 2000 to 2016 showed that entrepreneurial activity had negative spatial spillover effects on neighboring regions [19]. These phenomena can be attributed to–the siphoning effect of entrepreneurial activity. The siphoning effect occurs when a region attracts resources from neighboring regions, making itself more attractive and causing a decline in the attractiveness of surrounding neighboring regions [42]. When a region becomes increasingly active in entrepreneurship, it will attract capital, talent and other resources from neighboring regions, which will activate the siphoning effect [43], leading to the loss and lack of resources related to entrepreneurship in neighboring regions, ultimately inhibiting entrepreneurial activities [44]. This phenomenon is referred to as ’congesting’ factor resulting from inter-regional competition for entrepreneurial resources [45]. Based on the above discussion, the following hypothesis is proposed:

**H2.** Entrepreneurial activity in one city can have negative spillover effects on entrepreneurial activity in neighboring cities.

**Fig 1:**
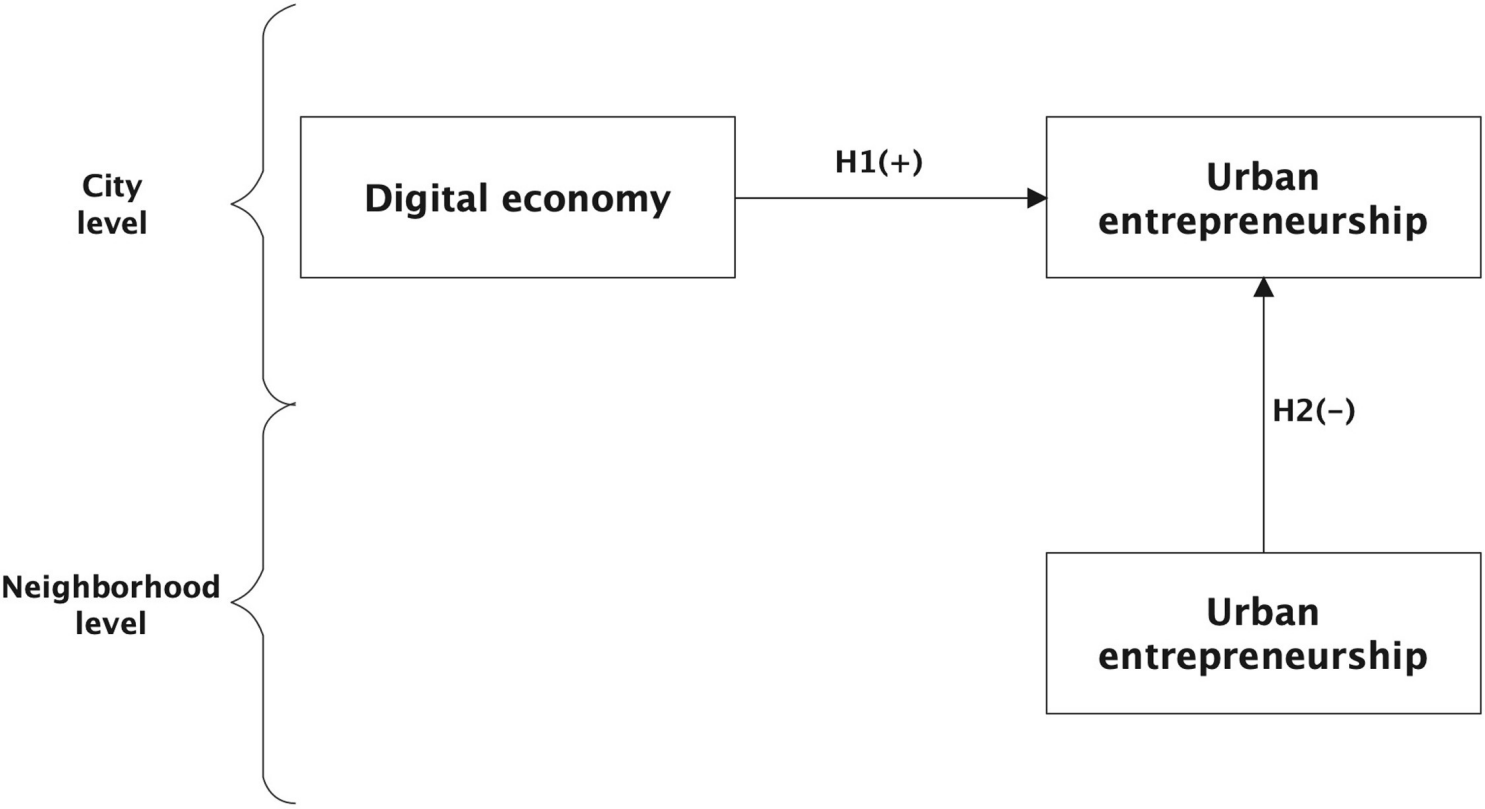
Analytical framework.

The impact of the digital economy on urban entrepreneurship is presented in the analytical framework shown in Fig 1. This analytical framework consists of two levels: the city level and the neighborhood level. At the city level, the growth of a city’s digital economy promotes the growth of entrepreneurship in that city. At the city level, the growth of a city’s digital economy promotes the growth of entrepreneurship in that city. It can be seen that urban entrepreneurship is affected by factors in the city, but also by factors in neighboring cities. This is an increasingly emerging area of urban entrepreneurship research, but one that has so far been neglected by the vast majority of scholars.

## 3. Data, variables and method

In this study, panel data of Chinese cities at the prefecture level from 2012 to 2019 are selected as research samples. The reason for closing the study period to 2019 is because of the outbreak of COVID-19 in 2020. The outbreak of COVID-19 caused millions of deaths globally and created a severe crisis in global economic growth, with forced business interruptions and a sharp drop in demand, leading many businesses to permanently exit the market. This is an extremely unique and unusual time for entrepreneurship. Therefore, this study does not include the period of the COVID-19 epidemic in the scope of the study. To ensure data availability and the robustness of the research results, any missing or abnormal values in the original data are eliminated. There are more than 300 prefecture-level administrative regions in China now, however, in 2012 there were only 285 prefecture-level cities in China. Deducting the four cities under the direct jurisdiction of the central government, that leaves 281 prefecture-level cities. After removing the missing and abnormal values, there are 252 prefectural cities, accounting for 90% of the 281 prefectures. A balanced panel of these 252 prefectural cities from 2012 to 2019 is created, which is the data used in this study. There is a total of 2,016 valid observations, covering 252 cities at the prefecture level. The data for relevant variables are obtained from the China Urban Statistical Yearbook for the respective years. The number of new private enterprises at the city level is obtained from the *Qixinbao* database, which uses the National Enterprise Credit Information Publicity System as a data source. *Qixinbao* is a fast enterprise information query tool for people in various industries to provide a wealth of enterprise information retrieval query services, including enterprise registration, investment bidding information, justice involved in lawsuits, qualifications, licenses, penalties and other enterprise-related information. The data on start-ups obtained through this channel mainly include information such as enterprise name, registered location, time of registration, registered capital, main business, and current operating status. This study utilizes *Qixinbao*’s advanced search to select regions and years to count the number of new registrations in each city, and organizes the search results into balanced panel data. These data are then integrated at the city level based on the registered locations of the start-ups.

### 3.1 Entrepreneurship

Existing studies commonly employ two main methods to measure entrepreneurial activity in a city or region: demographic and ecological methods. The demographic method uses the population of a city or region as the standardized base to measure entrepreneurial activity [46]. However, the ecological method uses the number of enterprises as the standardized base [29]. The use of demographic method to measure entrepreneurial activity is widely practice and has been adopted in previous studies [47, 48]. In this study, the demographic method is used specifically by measuring the number of new private enterprises per 10,000 people in the city. This measure is denoted by the symbol of *ENT*. The natural logarithmic of values of this measure are used in this study. By using the demographic method, the study partially addresses the problem of measurement bias that can arise from the heterogeneity of enterprise size and population size in the region [25].

### 3.2 Digital economy

Drawing on previous studies [49, 50], this study constructs an indicator system for the development of the digital economy in Chinese cities from two perspectives: digital inclusive financial development and Internet development. Among them, digital inclusive financial development is measured by a digital inclusive financial index [51]. This index captures the level of inclusive financial development in a city. For the measurement of Internet development, four indicators are used based on the research by Xu, Yang [52]: the number of Internet broadband access users per 100 people, the number of cell phone users per 100 people, the total amount of telecommunication services per capita, and the proportion of employees in the information transmission and computer software industries to the total number of employees. Additionally, following the approach of Xin, Chang [53], the weights of each variable are obtained using the entropy weight method, and the digital economy development index *DE* for each city is calculated. In this study, the natural logarithmic values are used. In the robustness test, a principal component analysis is used to obtain the weights of the variables, which are used to calculate the level of digital economy development of the city.

### 3.3 Covariates

The industrial structure, GDP per capita, and number of households can have an impact on entrepreneurial activity [54]. This study refers to the practice used by Hui, Zhao [55], specifically, the value added of the tertiary sector as a share of the GDP is considered as a variable of interest. In this study, a variable denoted by the symbol *SER* is introduced. Furthermore, the study incorporates GDP per capita (*GDP*) as it is known to be associated with entrepreneurial activity [56]. Additionally, the study includes controls for the number of households (*POP*), which is operationally defined as the natural logarithm of the number of households (10,000). Local human resources (*STU*) are also controlled for, which are operationally defined as the natural logarithm of the number of students enrolled in general undergraduate programs. Financial development (*FIN*) is measured by dividing the balance of loans from financial institutions at the end of the year by GDP. Moreover, the study measures the share of expenditure on education (*EDU*) by dividing the expenditure on education by the general budgetary expenditure of the local treasury. Similarly, the share of expenditure on science (*SCI*) is measured by dividing the expenditure on science by the general budgetary expenditure of the local treasury. Information about these variables is summarized in Table 1.

**Table 1:** Basic information and descriptive statistics for variables.

Symbol	Variable	Operational definition	Mean	Std. dev.	Min	Max
*ENT*	Entrepreneurial activity	Natural logarithm of new enterprises per 10,000 people	4.584	0.560	2.791	6.851
*DE*	Digital economy	Natural logarithm of digital economy index for cities	-2.283	0.513	-3.971	-0.389
*SER*	Industrial structure	The value added of tertiary sector as a share of GDP	41.844	9.934	11.470	83.520
*GDP*	GDP per capita	Natural logarithm of GDP per capita	10.738	0.550	9.007	12.456
*POP*	Total households	Natural logarithm of the total number of households	4.795	0.666	2.079	7.152
*STU*	Human resources	Natural logarithm of number of students enrolled in general undergraduate programs	10.639	1.291	7.139	13.958
*FIN*	Financial development	Loan balances of financial institutions as a share of GDP	1.026	0.644	0.118	9.622
*EDU*	Education expenditure	Education expenditure as a share of local fiscal expenditure	0.177	0.040	0.036	0.356
*SCI*	Science expenditure	Science expenditure as a share of local fiscal expenditure	0.017	0.016	0.001	0.207

### 3.4 Method

With the rapid development of intercity [57], the correlation between regions in terms of entrepreneurship cannot be ignored. The degree of entrepreneurship development in a city is often correlated with the degree of entrepreneurship development in other cities in its neighborhood. This is the spatial spillover of urban entrepreneurship. In empirical tests, this spatial spillover is tested by examining the significance of the regression coefficients of the spatial lag terms of the target variables. Therefore, here the spatial lag of urban entrepreneurship is included as an explanatory variable to explore the spatial spillover effect of urban entrepreneurship.

To address the potential endogeneity problem associated with the spatial lag term, existing studies typically introduce quantile regression into spatial econometric models [58]. Correspondingly, spatial panel quantile models containing the spatial lag term of urban entrepreneurship are constructed here to analyze the impact of the digital economy on entrepreneurial activities in different tiers of cities [59].

The spatial quantile regression model is a statistical method based on spatial data, and its basic principle is to introduce the concepts of spatial autocorrelation and quantile regression on the basis of the traditional linear regression model [60]. Among them, spatial autocorrelation refers to the existence of certain similarity or correlation between spatially neighboring areas; while quantile regression is a nonparametric regression method that can better deal with the distribution of data. Spatial quantile regression model is a new type of spatial data analysis method with strong application advantages [61]. It can provide scientific basis and decision support for decision makers in the fields of urban economic research, regional development planning and natural resources management [62]. With the widespread use of panel data, spatial panel quantile regression has emerged [63]. Combining spatial quantile regression with panel data and estimating the parameters of panel data variables using spatial quantile regression not only controls the heterogeneity of individuals to alleviate the endogeneity problem caused by omitted variables, but also analyzes the marginal effect of the independent variable on the dependent variable at a particular quantile as well as the spatial spillover effect, which helps to carry out the empirical analysis from multiple dimensions [58].

In terms of specific methods of estimation, following the method by Chen, Chen (63), this study adopts instrumental variable quantile regression (IVQR) estimation for the spatial panel quantile regression. The principle of IVQR estimation and its estimation steps are described in detail in some references such as Chernozhukov and Hansen [64], Autor, Houseman [65] and Chang, Wen [66]. The specific model setting is as follows:

$$\begin{array}{c} ENT_{it}=\alpha_{0\tau }+\rho_{\tau }W\times ENT_{it}+\beta_{1\tau }DE_{it}+\beta_{2\tau }SER_{it}+\beta_{3\tau }GDP_{it}+\beta_{4\tau }POP_{it}+\beta_{5\tau }STU_{it}+\\ \beta_{6\tau }FIN_{it}+\beta_{7\tau }EDU_{it}+\beta_{8\tau }SCI_{i}+\varepsilon_{it} \end{array}$$

In this study, *τ* represents the corresponding quantile points which are set as 0.2, 0.4, 0.6 and 0.8, respectively. The spatial weight matrix *W* used in this study is the spatial inverse distance weight matrix. The weight between two cities is calculated as the reciprocal of the distance between them [67]. *i* = 1, ⋯, 252; *t* = 2012, ⋯, 2019. *ε*_*it*_ is the random perturbation term.

## 4. Results

### 4.1 Baseline results

Table 2 presents the IVQR estimation results for the spatial panel quantile regression. According to Table 2, the regression coefficients of the digital economy are significantly positive for different quartile cases and gradually increase with an increase in quartiles. This indicates a positive correlation between the level of digital economy development in a city; and its entrepreneurial activity. Furthermore, it suggests that the role of the digital economy in promoting entrepreneurship becomes greater as the city’s entrepreneurship becomes more active. These findings align with previous studies [10, 13]. The economies of scale and consumer expansion effects generated by the digital economy provide more opportunities for entrepreneurs [11].

**Table 2:** Empirical results of baseline models.

	(1)	(2)	(3)	(4)
	0.2	0.4	0.6	0.8
	quantile	quantile	Quantile	quantile
*Wy*	-0.067^***^	-0.055^***^	-0.059^***^	-0.053^**^
	(0.023)	(0.02)	(0.02)	(0.023)
*DE*	0.073^*^	0.094^***^	0.11^***^	0.133^***^
	(0.038)	(0.033)	(0.033)	(0.038)
*SER*	0.013^***^	0.013^***^	0.014^***^	0.018^***^
	(0.002)	(0.001)	(0.001)	(0.002)
*GDP*	0.523^***^	0.544^***^	0.571^***^	0.506^***^
	(0.031)	(0.027)	(0.027)	(0.031)
*POP*	0.007	0.017	0.047^**^	0.011
	(0.024)	(0.021)	(0.021)	(0.024)
*STU*	-0.056^***^	-0.068^***^	-0.063^***^	-0.055^***^
	(0.015)	(0.013)	(0.013)	(0.015)
*FIN*	0.161^***^	0.167^***^	0.151^***^	0.109^***^
	(0.023)	(0.02)	(0.02)	(0.023)
*EDU*	-1.748^***^	-1.059^***^	-0.810^***^	-0.726^**^
	(0.291)	(0.255)	(0.255)	(0.291)
*SCI*	2.721^***^	2.794^***^	2.516^***^	5.904^***^
	(0.826)	(0.722)	(0.724)	(0.824)
_cons	-0.702^*^	-0.824^**^	-1.168^***^	-0.397
	(0.402)	(0.352)	(0.352)	(0.401)
*Obs*	2016	2016	2016	2016
*N*	252	252	252	252

Standard errors in parentheses

^*^ *p* < 0.1

^**^ *p* < 0.05

^***^ *p* < 0.01.

The digital economy has bred entrepreneurial opportunities in cities from both the demand side and the supply side. From the demand side, the development of the digital economy has promoted the development of new consumer businesses such as live e-commerce and smart business districts [68], and stimulated consumers’ demand for product diversification [69], which further promotes the development of the product market, thus providing more opportunities for entrepreneurial activities in cities. From the supply side, digital information consumption can transform the technology-economy paradigm of traditional industries and improve the success rate of urban entrepreneurship by increasing production efficiency and enhancing the coordination of production factors of enterprises [70]. Clearly, the results of this study provide new empirical evidence on the synergistic role of active governments and efficient markets in helping digital economy policies to stimulate urban entrepreneurship.

The regression coefficients for the spatially lagged terms of the dependent variable are significantly negative in all four quartiles in Table 2. This finding suggests that urban entrepreneurship has a significant siphoning effect when other multivariate factors are considered [42]. The development of entrepreneurship in one city can have a heterogeneous effect on entrepreneurship in neighboring cities [18]. The strong attraction of a city to neighboring cities, where entrepreneurial activities are more active due to specific resource location advantages, can attract investment, consumption, or resources from neighboring cities, thus slowing the development of entrepreneurship in the city [17]. This siphoning effect illustrates the resource-dependent nature of entrepreneurship [71].

### 4.2 Robustness analysis

To test the robustness of the findings, the calculation of the digital economy index was changed from the previous entropy weight method to the principal component method. The summarized results are in Table 3. According to Table 3, the effects of the digital economy on entrepreneurship are positive and increase with increasing quartiles. However, they are not significant when the quartiles are 0.2 and 0.4, and only become significant at the 0.6 and 0.8 quartiles. This suggests that the impact of the digital economy on entrepreneurship is not significant when cities have lower levels of entrepreneurial activity. The digital economy has a catalytic effect on entrepreneurship, but only when urban entrepreneurship is more active. This suggests that the digital economy’s role in promoting urban entrepreneurship is context-dependent and relies on specific entrepreneurial conditions [72, 73]. The results in Table 3 are broadly consistent with those in Table 2 at the quartiles of 0.6 and 0.8. Additionally, the regression coefficients from the spatial lag terms of the dependent variable are significantly negative for all quartiles, which aligns with the results in Table 2.

**Table 3:** Empirical results after changing the measurement of the independent variable.

	(1)	(2)	(3)	(4)
	0.2	0.4	0.6	0.8
	quantile	quantile	Quantile	quantile
*Wy*	-0.073^***^	-0.063^***^	-0.06^***^	-0.052^**^
	(0.023)	(0.02)	(0.02)	(0.023)
*DE*	0.013	0.147	0.885^***^	1.653^***^
	(0.329)	(0.288)	(0.287)	(0.329)
*SER*	0.0131^***^	0.013^***^	0.0137^***^	0.018^***^
	(0.002)	(0.001)	(0.001)	(0.002)
*GDP*	0.548^***^	0.579^***^	0.587^***^	0.51^***^
	(0.029)	(0.025)	(0.025)	(0.029)
*POP*	-0.001	0.01	0.04^*^	0.007
	(0.024)	(0.021)	(0.021)	(0.024)
*STU*	-0.051^***^	-0.062^***^	-0.067^***^	-0.052^***^
	(0.015)	(0.013)	(0.013)	(0.015)
*FIN*	0.171^***^	0.186^***^	0.154^***^	0.097^***^
	(0.023)	(0.02)	(0.02)	(0.023)
*EDU*	-1.795^***^	-0.995^***^	-0.829^***^	-0.773^***^
	(0.294)	(0.257)	(0.256)	(0.294)
*SCI*	2.939^***^	3.08^***^	2.988^***^	5.27^***^
	(0.837)	(0.731)	(0.729)	(0.836)
_cons	-1.142^***^	-1.395^***^	-1.068^***^	0.126
	(0.428)	(0.374)	(0.373)	(0.428)
*Obs*	2016	2016	2016	2016
*N*	252	252	252	252

Standard errors in parentheses

^*^ *p* < 0.1

^**^ *p* < 0.05

^***^ *p* < 0.01.

The use of different spatial weighting matrices can have an impact on the results [74]. In this study, the original spatial weight matrix was replaced with a new spatial weight matrix constructed based on the proximity criterion of whether two cities are in the same province. If two cities are in the same province, the weight is 1; if not, the weight is 0. This proximity-based spatial weight matrix consists of 1s and 0s. The regression results obtained after applying this new spatial weight matrix are presented in Table 4. According to Table 4, the regression coefficients for the digital economy are insignificant at the 0.2 quantile. However, they are significantly positive at the 0.4, 0.6, and 0. 8 quantiles, and the magnitude of the coefficient increases with the increase in the quantile. This means that the results in Table 4 are consistent with those in Table 2, except for the 0.2 quantile, where the promotion of entrepreneurship by the digital economy is not significant. Furthermore, the regression coefficients for the spatial lag terms of the dependent variable are significantly negative in all quartiles, which aligns with the results in Table 2.

**Table 4:** Empirical results after replacing the spatial weight matrix.

	(1)	(2)	(3)	(4)
	0.2	0.4	0.6	0.8
	quantile	quantile	Quantile	quantile
*Wy*	-0.003^***^	-0.003^***^	-0.007^***^	-0.006^***^
	(0.001)	(0.001)	(0.001)	(0.001)
*DE*	-0.043	0.795^***^	1.167^***^	1.797^***^
	(0.32)	(0.281)	(0.284)	(0.332)
*SER*	0.013^***^	0.012^***^	0.013^***^	0.018^***^
	(0.002)	(0.001)	(0.001)	(0.002)
*GDP*	0.55^***^	0.557^***^	0.568^***^	0.515^***^
	(0.029)	(0.025)	(0.026)	(0.03)
*POP*	-0.017	0.009	0.0434^**^	0.007
	(0.023)	(0.021)	(0.021)	(0.024)
*STU*	-0.059^***^	-0.074^***^	-0.07^***^	-0.047^***^
	(0.014)	(0.013)	(0.013)	(0.015)
*FIN*	0.188^***^	0.185^***^	0.159^***^	0.076^***^
	(0.023)	(0.02)	(0.02)	(0.023)
*EDU*	-1.531^***^	-1.094^***^	-1.130^***^	-0.97^***^
	(0.291)	(0.256)	(0.259)	(0.302)
*SCI*	1.272	1.681^**^	1.333^*^	4.463^***^
	(0.789)	(0.694)	(0.702)	(0.82)
_cons	-1.191^***^	-0.680^*^	-0.621	0.226
	(0.427)	(0.376)	(0.380)	(0.444)
*Obs*	2016	2016	2016	2016
*N*	252	252	252	252

Standard errors in parentheses

^*^ *p* < 0.1

^**^ *p* < 0.05

^***^ *p* < 0.01.

The results of this study may vary depending on the selected time span [75]. To address this concern, a new time span from 2014 to 2019 was randomly selected, and the regression results are presented in Table 5. The regression coefficients for the digital economy under the new time span are positive and increase with the quantile; however, they are only significant at the quantiles of 0.6 and 0.8. This means that the results of the study are consistent with the results in Table 2 at quartiles of 0.6 and 0.8. Furthermore, the regression coefficients for the spatial lag terms of the dependent variable are significantly negative in all quartiles, which aligns with the results in Table 2.

**Table 5:** Empirical results from 2014 to 2019.

	(1)	(2)	(3)	(4)
	0.2	0.4	0.6	0.8
	quantile	quantile	Quantile	quantile
*Wy*	-0.08^***^	-0.076^***^	-0.065^***^	-0.055^**^
	(0.026)	(0.023)	(0.022)	(0.026)
*DE*	0.235	0.475	1.146^***^	2.184^***^
	(0.357)	(0.316)	(0.314)	(0.357)
*SER*	0.012^***^	0.01^***^	0.01^***^	0.012^***^
	(0.002)	(0.002)	(0.002)	(0.002)
*GDP*	0.624^***^	0.584^***^	0.549^***^	0.479^***^
	(0.034)	(0.03)	(0.03)	(0.034)
*POP*	-0.024	-0.007	0.003	-0.019
	(0.027)	(0.024)	(0.023)	(0.027)
*STU*	-0.036^**^	-0.033^**^	-0.037^**^	-0.02
	(0.017)	(0.015)	(0.015)	(0.017)
*FIN*	0.146^***^	0.167^***^	0.157^***^	0.094^***^
	(0.024)	(0.021)	(0.021)	(0.024)
*EDU*	-0.523	-0.144	-0.091	-0.147
	(0.335)	(0.296)	(0.294)	(0.334)
*SCI*	2.708^***^	3.058^***^	3.805^***^	4.457^***^
	(0.848)	(0.75)	(0.745)	(0.848)
_cons	-1.942^***^	-1.363^***^	-0.601	0.74
	(0.490)	(0.433)	(0.431)	(0.49)
*Obs*	1512	1512	1512	1512
*N*	252	252	252	252

Standard errors in parentheses

^*^ *p* < 0.1

^**^ *p* < 0.05

^***^ *p* < 0.01.

### 4.3 Heterogeneity analysis

Economic and social phenomena, in general, exhibit significant regional heterogeneity [76]. According to China’s National Development and Reform Commission (NDRC), China can be divided into east, center and west, a division that is mainly policy-based [77]. The eastern part of the country refers to the provinces that were the first to implement the coastal opening policy and have a high level of economic development; the central part refers to the less economically developed regions, while the western part refers to the less economically developed western regions. Specifically, the eastern region includes Beijing, Tianjin, Shanghai, Liaoning, Jiangsu, Zhejiang, Fujian, Shandong, and Guangdong; the central region includes Hebei, Shanxi, Jilin, Heilongjiang, Anhui, Jiangxi, Henan, Hubei, Hunan, and Hainan; and the western region includes Inner Mongolia, Guangxi, Chongqing, Sichuan, Guizhou, Yunnan, Tibet, Shaanxi, Gansu, Qinghai, Ningxia, and Xinjiang.

Separate estimations are performed for the eastern, central, and western samples, and the results are summarized in Table 6. According to Table 6, in the eastern region, the promotion of entrepreneurship by the digital economy is significant only in the 0.8 quartile. In the Central region, the contribution of the digital economy to entrepreneurship is significant in all four quartiles, but relatively weakest in the 0.4 quartile. In the western region, the contribution of the digital economy to entrepreneurship is significant only in the 0.4 and 0.6 quartiles. Additionally, the siphoning effect of entrepreneurial activity on neighboring cities is significant only at the 0.8 quantile in the Eastern region and only at the 0.2 quantile in the central region. However, in the western region, it is significant at all quantiles. As can be seen, regional heterogeneity is evident in terms of the impact of the digital economy on entrepreneurship activity.

**Table 6:** Results of the test for regional heterogeneity.

	East				Central				West			
	(1)	(2)	(3)	(4)	(5)	(6)	(7)	(8)	(9)	(10)	(11)	(12)
	0.2	0.4	0.6	0.8	0.2	0.4	0.6	0.8	0.2	0.4	0.6	0.8
	quantile	quantile	quantile	quantile	Quantile	quantile	quantile	quantile	Quantile	quantile	quantile	quantile
*Wy*	-0.061	-0.032	-0.034	-0.108^**^	-0.046^*^	-0.012	-0.013	0.003	-0.144^***^	-0.174^***^	-0.232^***^	-0.212^***^
	(0.048)	(0.041)	(0.041)	(0.052)	(0.026)	(0.023)	(0.023)	(0.025)	(0.042)	(0.037)	(0.04)	(0.043)
*DE*	-0.382	-0.268	0.609	1.298^**^	1.712^**^	1.492^**^	1.641^***^	1.699^**^	0.474	2.374^***^	2.455^***^	1.306
	(0.467)	(0.404)	(0.402)	(0.512)	(0.719)	(0.62)	(0.618)	(0.692)	(0.801)	(0.714)	(0.76)	(0.837)
*VC*	Yes	Yes	Yes	Yes	Yes	Yes	Yes	Yes	Yes	Yes	Yes	Yes
*Obs*	832	832	832	822	768	768	768	768	416	416	416	416
*N*	104	104	104	104	96	96	96	96	52	52	52	52

VC refers to the controlled variables; standard errors are in parentheses

^*^ *p* < 0.1

^**^ *p* < 0.05

^***^ *p* < 0.01.

Regional development policies, which have various impacts, are a significant factors contributing to heterogeneity [78]. In China, the Yangtze River Economic Belt (YREB) refers to the economic circle near the Yangtze River, comprising 11 provinces, including Shanghai, Jiangsu, Zhejiang, Anhui, Jiangxi, Hubei, Hunan, Chongqing, Sichuan, Yunnan and Guizhou [79]. The remaining provinces are non-YREB areas. The YREB region covers an area of approximately 2,052,300 square kilometers, or 21.4% of China’s land area, and has more than 40% of China’s population and GDP. In September 2016, the Outline of the Development Plan for the Yangtze River Economic Belt was officially issued, establishing the development pattern of the YREB. As the regional integration and development strategy of the YREB continues to strengthen, it has resulted in policy differences between the YREB and non-YREB regions. Separate estimations are performed for the YREB and non-YREB samples, and the results are summarized in Table 7. According to Table 7, the promotion of entrepreneurial activity by the digital economy is significant only in the 0.6 and 0.8 quartiles in YREB. However, in the non-YREB regions, it is significant in all quartiles. The siphoning effect of entrepreneurial activity is significant in all quartiles in the YREB; while it is significant only at the 0.2 quartile in the non-YREBs. These findings highlight the presence of policy-relevant heterogeneity, indicating that the impact of regional development policies differ between the YREB and non-YREB areas.

**Table 7:** Results of the test for policy-relevant heterogeneity.

	YREB				non-YREB			
	(1)	(2)	(3)	(4)	(5)	(6)	(7)	(8)
	0.2	0.4	0.6	0.8	0.2	0.4	0.6	0.8
	quantile	quantile	quantile	quantile	quantile	quantile	quantile	quantile
*Wy*	-0.098^***^	-0.082^**^	-0.086^***^	-0.078^**^	-0.046^*^	-0.03	-0.023	-0.026
	(0.037)	(0.033)	(0.033)	(0.037)	(0.028)	(0.023)	(0.023)	(0.028)
*DE*	0.0516	0.055	0.111^**^	0.191^***^	0.116^**^	0.138^***^	0.158^***^	0.151^***^
	(0.062)	(0.055)	(0.055)	(0.062)	(0.049)	(0.041)	(0.041)	(0.049)
*VC*	Yes	Yes	Yes	Yes	Yes	Yes	Yes	Yes
*Obs*	824	824	824	824	1192	768	768	768
*N*	103	103	103	103	149	96	96	96

VC refers to the controlled variables; standard errors are in parentheses

^*^ *p* < 0.1

^**^ *p* < 0.05

^***^ *p* < 0.01.

## 5. Discussion

### 5.1 Theoretical implications

Firstly, This study fills a gap in the existing literature by examining the impact of the digital economy on urban entrepreneurial activity across cities with varying levels of entrepreneurial activity [36, 37]. Previous research has emphasized the role of the digital economy in driving entrepreneurship, arguing that its growth will inevitably lead to increased entrepreneurial activity [9, 10]. However, in cities with lower levels of entrepreneurial activity, the facilitating effect of the digital economy on entrepreneurship may not be significant. In other words, the impact of the digital economy on entrepreneurship is contingent upon specific context in which it occurs. Although, some of the previous literature suggests that the digital economy promotes entrepreneurship [10, 38, 80], this existing literature generally ignores localized characteristics at different levels of entrepreneurship. The results of this study show that the impact of the digital economy on urban entrepreneurship can vary with the level of entrepreneurship quartile, therefore, the localized characteristics of the respondent variable at each quartile can be effectively observed, which makes up for the shortcomings of the existing references. Thus, this study effectively contributes to the existing literature by explaining the impact of digital economy on entrepreneurship from a contingency theory perspective enriching the understanding of this subject [11, 13].

Secondly, this study extends the knowledge spillover theory of entrepreneurship by exploring and testing the siphoning effect of urban entrepreneurial activity effectively [16, 72]. While the knowledge spillover theory emphasizes the positive spatial spillover effects of entrepreneurial activity, it has faced skepticism [15, 39, 40]. Although the siphon effect occurs repeatedly in economic systems [42, 81], there is a lack of research on the siphon effect in the field of entrepreneurship. This study shows that urban entrepreneurial activity generates negative spatial spillover effects on entrepreneurial activity in neighboring cities, indicating is the presence of a siphoning effect [43]. These findings align with some previous studies [17, 19] and shed light on the competition between neighboring cities for entrepreneurship-related resources. Therefore, this study complements the knowledge spillover theory by adopting a resource dependence perspective [44, 71].

Lastly, this study conducts a thorough heterogeneity analysis of the impact of the digital economy on entrepreneurship, uncovering the relevant impact mechanisms and variations. This analysis enhances the assessment of entrepreneurship promotion policies and offers valuable insights for targeted development of digital economy to stimulate entrepreneurial activities [82, 83]. The results show that in the Eastern region, the digital economy significantly promotes entrepreneurship only in the 0.8 quartile. In the YREB, the digital economy’s impact on entrepreneurship activity is significant only in the 0.6 and 0.8 quartiles. These findings provide an important basis for decision-making in leveraging the digital economy to foster entrepreneurial tendencies on a larger scale [3, 4].

### 5.2 Practical implications

Based on the differentiation of urban entrepreneurial activities, this study proposes several suggestions: First, it is crucial to recognize the heterogeneity in the impact of the digital economy on cities with varying levels of entrepreneurial activity. Differentiated measures and strategies should be adopted for the development of the digital economy in cities at different levels of entrepreneurial activity. For example, cities with lower levels of entrepreneurial activity, may need to focus on supplementing the development of the digital economy with the growth on the tertiary and financial industries and increasing investment in scientific research. Secondly, there is a need to expedite the construction of city clusters and build metropolitan circles with the central city as the core. Finally, it is important to consider the degree of entrepreneurial activity in the city, its location, policy conditions, economic volume, and population density to formulate targeted and precise entrepreneurship promotion policies and thus ensure their effectiveness.

### 5.3 Research limitations and future research directions

Because this study focuses on entrepreneurship in a normative setting, its study period ends in 2019 and does not include the COVID-19 outbreak period from 2020 to 2023. Although, dramatic external shocks such as the COVID-19 epidemic are very rare, exploring the impact of major shocks in the external environment on entrepreneurship remains a topic worth digging deeper into. The sudden COVID-19 epidemic in 2020 brought a severe crisis to the global economy, with almost all major countries in the world except China experiencing negative economic growth. A large number of companies were forced out of the market, which had a chilling effect, leaving a large number of potential entrepreneurs in a wait-and-see and waiting mode. Thus, it can be expected that the COVID-19 epidemic inhibited entrepreneurship. However, this needs to be tested with empirical data. This is a direction for the next research.

## 6. Conclusion

This study adopts a resource dependence perspective to examine the impact of the digital economy on urban entrepreneurial activities at different quantile levels. A spatial panel quantile regression model is built using panel data from 252 prefecture-level cities from 2012 to 2019. The results suggest that the digital economy significantly promotes urban entrepreneurship. However, this finding is robust at high quantile levels and may not be significant at low quantile levels. Additionally, there is a siphoning effect of entrepreneurial activity in cities, meaning that the development of entrepreneurship in one city can dampen entrepreneurial activity in neighboring cities. Moreover, the impact of the digital economy on urban entrepreneurship and the siphoning effect varies across regions and policy. This highlights the heterogeneity in the relationship between digital economy and urban entrepreneurship.
